# Developmental screening in a Canadian First Nation (Mohawk): psychometric properties and adaptations of ages & stages questionnaires (2nd edition)

**DOI:** 10.1186/1471-2431-14-23

**Published:** 2014-01-28

**Authors:** Carmen Dionne, Suzie McKinnon, Jane Squires, Jantina Clifford

**Affiliations:** 1 Canadian Research Chair on Early Intervention, Department of Psychoeducation, Université du Québec à Trois-Rivières, P.O. Box 500, Trois-Rivières, Quebec G9A 5H7, Canada; 2 Department of Psychoeducation, Université du Québec à Trois-Rivières, Trois-Rivières, Canada; 3 Early Intervention Program, Center for Excellence in Developmental Disabilities, University of Oregon, Eugene, USA; 4 Early Intervention Program, Department of Special Education, University of Oregon, Eugene, USA

**Keywords:** Children, First nation, Developmental delay, Screening, ASQ

## Abstract

**Background:**

The need for early intervention tools adapted to the First Nation culture is well documented. However, standards derived from First Nation communities are absent from the literature. This study examines the psychometric properties of an adaptation of a caregiver-completed screening tool, the *Ages & Stages Questionnaires* (ASQ), for the Mohawk population.

**Methods:**

Participants who completed the questionnaires include 17 teachers, along with the parents of 282 children (130 girls and 152 boys) between the ages of 9 and 66 months who attend the Child and Family Center Mohawk Territory, Quebec.

**Results:**

For the internal consistency of the four questionnaires (36-, 42-, 48- and 54-month intervals), Cronbach’s alphas varied between .61 and .84. Five results were below 0.60: “gross motor” (Q36 and Q42), “problem solving” (Q36) and “personal-social” (Q36 and Q42). A comparison of the results shows that parents and teachers agreed in 85% of the cases concerning the referral of the child for further evaluation. Moreover, the group discussion with the parents revealed that the use of the questionnaire was appreciated and was deemed appropriate for use within the community.

**Conclusion:**

The results show that the ASQ is a screening test that may be appropriate for use with children from communities that are seemingly very different in terms of geographic, climatic and cultural backgrounds. This preliminary study with the Child and Family Center appears to support further study and the use of the ASQ with the Mohawk population.

## Background

The impact and importance of a young child’s early life experiences on all domains of development is well documented and supported by research in neuroscience and developmental psychology [1]. In addition, it has been demonstrated that quality early intervention significantly influences the lives of children with developmental disabilities and reduces the impact of these difficulties on family and social networks [2].

Parents have been found to be accurate assessors of their child’s early development, when asked about current, observable behavior [3]. Parent involvement in both the assessment and intervention process is one essential component of quality early intervention programs that has been clearly identified as best practices in early intervention [4]. A review of recommended practices in early intervention conducted by Sandall, Hemmeter, Smith, and McLean [5] suggests that it is important to use an approach where families and caregivers of young children participate and contribute during the assessment and planning processes prior to implementing intervention services. Families and caregivers can then collaborate with interventionists and play an important role in the identification and development of the goals and objectives to be targeted in their child’s individualized plan. During this process, parents share formal and informal information that can help the team make choices and informed decisions. In this vein it is also important to select interventions and resources that capitalize on parents’ existing skills while further developing their abilities and building their trust. Of course, these interventions must take into consideration the family’s culture and language, as well as other characteristics of their community in which they live.

The first step in the early intervention process is identifying young children whose developmental skills are not typical and may be in need of individualized and focused assistance. However, in spite of accumulated evidence supporting early identification [6] and intervention [7,8], screening for developmental difficulties remains problematic, and significant delays are often unidentified until children enter kindergarten [9].

The challenges related to screening are even more significant for young First Nation children. The use of available screening tools with First Nation children raises numerous issues. First, there are few studies pertaining to early childhood that directly address this population [10]. Second, there is a lack of research and information on how culture may influence the results of evaluation tools when used within First Nation communities. The appropriateness and effectiveness of evaluation efforts must be examined before they are implemented to determine if the approaches are in line with the needs, interests, developmental expectations, and learning styles of the First Nation population.

According to Hernandez [11], most of the existing standardized tests are not developed with adequate consideration of cultural diversity. Many First Nation parents and caregivers working with early childhood programs believe that formal tools used to support non-Native children and their families are not culturally appropriate or even helpful for their children [12,13].

Moreover, assessment tools that are not culturally appropriate may result in negative consequences for young native children such as under- or over-referral [14]. It is of critical importance that assessment tools be culturally adapted in order to yield valid results that minimize or eliminate under- or over-identification of children with difficulties [15]. So, how can the adequacy of an instrument for young children in First Nation communities be evaluated?

Many components are involved in determining whether a tool is culturally appropriate or not. Ball [9] emphasizes that instrument standardization should minimally include a sample of Indigenous populations. According to this Canadian researcher, there is an urgent need to establish a set of principles, methods, and tools in order to better evaluate the development of Native children and identify their needs [16].

Currently, there are a handful of valid and reliable instruments that are typically used with Native children by researchers, caregivers, and parents (e.g., The Work Sampling System, Ages & Stages Questionnaires, Nipissing Developmental Screen, Gesell, Battelle Developmental Inventory). However, even if the validity and reliability of these instruments have been established, many questions still remain regarding their use within First Nation communities, such as: Are test items, materials, or administration methods culturally biased? Were the normative standards established with the inclusion of Native people? What adaptations are required? When adjustments are made to reflect cultural differences are there effects on the validity of the results?

In a study undertaken by Dion-Stout & Jodoin [17] for *The Maternal & Child Health Program First Nations and Inuit Heath Branch*, Dion-Stout and Jodoin [17] did not find any tools specifically developed or adapted for First Nation populations. Ball [9] found that the *Ages* & *Stages Questionnaires* (ASQ) [18] is the most commonly used screening tool in First Nation early intervention programs in Western Canada and is the primary tool used by the First Nations of British Columbia. In their report Dion-Stout and Jodoin also recommended the use of the ASQ, as it can be readily adapted to reflect the day to day living situation and culture of many different populations, including First Nations.

The ASQ [18] is a parent/caregiver completed screening tool with excellent psychometric properties that has been successfully used with a variety of populations [19-21]. Survey results [4] indicate that it is user-friendly, that parents/caregivers generally enjoy completing it, and that they find the results helpful. Of the children in the ASQ normative sample used for validation in the U.S., 15% were Native American. However, amongst studies that have been done in Canada, no standards are currently available for First Nation populations.

The purpose of this study is to assess the relevance and usefulness of the ASQ for the parents of Mohawk children and to collect the data needed to evaluate the tool within this context. Usage of the ASQ was evaluated with a population of young children attending the Child and Family Center located on a First Nation Mohawk territory in Eastern Canada. The study has three objectives: the first is to present the internal consistency indices (Cronbach’s alpha, correlations and cut-off points); the second objective is to describe the agreement between parents and teachers concerning the referral of the child for further evaluation; the third objective is to explore whether the ASQ is culturally appropriate for the First Nation community.

## Method

### Participants

The participants who completed the questionnaires were 17 teachers, along with the parents of 282 Mohawk children (130 girls and 152 boys) between the ages of 9 and 66 months who attended the Child and Family Center during the years 2006–2009. These families live in a First Nation community in Mohawk Territory in Quebec. Eight parents participated in a focus group to discuss their opinions regarding the instrument. Subsamples of the data were used to satisfy different research objectives. For the first objective, ASQ results were examined only for children whose parents completed a 36-, 42-, 48- and 54-month questionnaire (Table 1). For the second objective, all children whose parents and teachers completed one questionnaire (10 to 60 months) were considered.

**Table 1:** Number of participants by research objective

**Objective**	**N**			
	**Children**	**Parents**	**Teachers**	**Questionnaires completed**
Objective 1 Age interval 36–54 months (parents only)	196	196	NA	258
Objective 2 Age interval 10–60 months (Paired parents and teachers)	266	266	17	788 (394 completed by parents and 394 completed by teachers)
Objective 3 Focus group	NA	8	NA	NA

Socio-demographic data were gathered on the families of 229 Mohawk children. Almost all parents were of Mohawk descent and the majority spoke English at home. The proportion of mothers who had a high school diploma was 18.6% and of the fathers, 27.1%. About 5% of mothers held a college degree compared with 7.3% for the fathers. The percentage of mothers having a university degree (9.5%) was much higher than the fathers (0.9%). Among the participating families, 37.2% had an annual income of less than $25,000, while 24.3% had an income between $25,000 and $40,000 and more than one third of the families had an annual income of over $40,000.

This study was approved by the research ethics committee of the Université du Québec à Trois-Rivières.

### Instruments

#### Ages & stages questionnaires

The ASQ [18] is a screening tool used to assess children’s development. Parents or practitioners who know the child well complete the questionnaire at one of 19 intervals (2nd edition), according to the age of the child (i.e., 4, 6, 8, 10, 12, 14, 16, 18, 20, 22, 24, 27, 30, 33, 36, 42, 48, 54 or 60 months). Each questionnaire is composed of 30 clearly, simply, and precisely formulated items, targeting abilities or behaviors that are milestone skills for the specific age range of the interval. These items are organized within five developmental domains: communication, gross motor, fine motor, problem solving, and personal-social. Parents/practitioners answer each item by observing the child and selecting either “yes” to indicate that the child demonstrates the ability described by the statement, “sometimes” to indicate that the skill is inconsistent or emerging, and “not yet” when the child has not yet shown evidence of manifesting the ability or behavior. Depending upon the selected responses, points are awarded to each answered item and total scores are compared with statistically derived cut-offs based on means and standard deviations to indicate whether the child appears to be developing typically, or whether he or she should be referred for a more comprehensive assessment. A “monitoring zone” was added to the third edition of the ASQ to assist in identifying children with domain scores that are “close to the cutoff” and may warrant further attention or developmental guidance.

Psychometric properties of the U.S. version were studied using over 8,000 questionnaires [3]. Data were reported on concurrent validity, test-retest reliability, and inter-rater reliability. Test-retest reliability, or the score comparison between two questionnaires completed by a caregiver (n = 175) at a two-week interval, was 94%. Inter-observer reliability, or the comparison of children’s classifications based on questionnaires completed by parents (n = 112) and professional examiners (n = 2), was also 94%. Concurrent validity, the percentage of agreement between classifications (e.g., “delayed” or “typically developing”) according to results from the ASQ and other standardized assessments, ranged from 76% for the 4-month ASQ to 91% for the 36-month ASQ. Sensitivity (i.e., the ability of the ASQ to correctly identify children experiencing delays) ranged from 51% for the 4-month ASQ to 90% for the 36-month ASQ. Overall sensitivity was 76%. Specificity (i.e., the ability of the ASQ to correctly identify typically developing children) ranged from 81% for the 16-month ASQ to 92% for the 36-month ASQ, with an overall specificity rate of 86%.

For the purpose of this study, the questionnaires were slightly modified. Some visual changes, and others linked to filling out the questionnaire according to the cultural norms were made prior to administration. For example, in terms of visuals, we added the logo of the Child and Family Center and inserted other Mohawk-derived graphics. In regards to the content, for the 36- to 42-month questionnaires, the communication item “Ask your child to put the shoe on the table” was modified. We suggested to parents that they could use any object—not necessarily a shoe—as we felt parents would find it unacceptable to ask a child to put a shoe on the table, and children might also be reluctant to do so if they had been taught otherwise.

#### Parent demographic questionnaire

A demographic questionnaire was sent to the parents requesting information on ethnicity, income level, and language spoken at home. It was accompanied by a consent form from the Child and Family Center.

### Procedures

Participants (both parents and teachers) read and signed an informed consent form describing the goal and objectives of the study, as well as the roles of both parent/teacher and child in the research.

Participants then completed an age-appropriate ASQ questionnaire for their child every 12 months (*M* = 12.53; *SD* = 3.28). A subsample of participating parents was asked to participate in a focus group. Each focus group lasted a minimum of three hours. Parents were selected in such a way as to have a mix that included children from different age groups, some with special needs, as well as some who were considered typically developing.

### Analysis

The research objectives were addressed using descriptive, correlational and reliability analyses (Cronbach’s alpha coefficients).

## Results

Objective 1 is to present indices of internal consistency (Cronbach’s alpha, correlations and cut-off points). For this analysis, we used Cronbach’s alphas by developmental domain and calculated the correlation coefficients.

The results of 258 questionnaires completed by parents for the 36-, 42-, 48- and 54-month intervals are presented (the internal consistency analyses pertain only to these age intervals because there were not enough data for the other questionnaires to ensure reliable statistical processing).

Table 2 shows Cronbach’s alphas for the Mohawk and U.S. populations by developmental domain for the four questionnaires. The U.S. results are presented as a guide to contrast with the Cronbach’s alpha for the Mohawk population. For the 36^th^ month questionnaire (Q36) results for Mohawk children show alpha values ranging from .70 to .79, with insufficient values for “gross motor” (.43), “problem solving” (.31), and “personal-social” (.40); for Q42, alpha values are acceptable, varying from .56 to .70, except for “personal-social”, which has an insufficient value of .31; in all of the questionnaires completed for older children the alpha coefficients were higher. For example, Q48, alpha values range from .70 to .84; and Q54 shows values ranging from .64 to .83.

**Table 2:** Standardized alphas by developmental domain and age interval for Mohawk and U.S. populations

**Age interval**	**n**		**Communication**		**Gross motor**		**Fine motor**		**Problem solving**		**Personal-social**	
	**Mo**	**U.S.**	**Mo**	**U.S.**	**Mo**	**U.S.**	**Mo**	**U.S.**	**Mo**	**U.S.**	**Mo**	**U.S.**
36 months^a^	68	231	.70	.69	.43	.76	.79	.72	.31	.66	.40	.55
42 months^b^	55	950	.68	.72	.56	.68	.61	.76	.70	.72	.31	.66
48 months^a^	74	336	.84	.79	.75	.84	.81	.86	.71	.85	.70	.86
54 months^b^	61	586	.83	.83	.71	.73	.73	.79	.64	.75	.65	.71

*Note.* Mo: Mohwak. Cronbach’s alphas for the U.S. sample were combined from two different sources: ^a^Squires et al. [3] (second edition) and ^b^Squires et al. [18] (third edition) user’s guides as the coefficients for the 42- and 54-month intervals were not available in the second edition. Coefficients for the U.S. sample are presented for informational purposes.

For Cronbach’s alphas below .60, an item deletion procedure was executed. Results showed increased coefficients for each domain, especially “gross motor” (Q36), which increased from .43 to .51; and “personal and social” (Q42), which increased from .31 to .46.

Table 3 presents Pearson’s correlation coefficients calculated based on the analysis of the developmental domains and total scores for the 36-, 42-, 48-, and 54-month questionnaires for the Mohawk and U.S. populations. Here also, the U.S. correlations are presented as a guide to contrast with those of the Mohawk population. The correlations for Mohawk children between the “communication”, “gross motor”, “fine motor”, “problem solving”, and “personal-social” developmental domains and the overall score of the four questionnaires varied from low to high (.46 to .87). Correlations for Q36 varied from .48 to .77. The correlations of “gross motor” (.48) and “problem solving” (.49) were low. Correlations of domains Q42 varied from .58 to .80. For Q48, correlations varied from good to very good (.74 to .87); for Q54, they were very good (.62 to .87), with the exception of “gross motor”, with a value of .46. In summary, the “communication”, “fine motor”, “problem solving”, and “personal-social” domains exhibited good coefficient correlations in relation to the overall scores of the four above-mentioned questionnaires (.73 to .87), with the exception of Q36 (problem solving). However, correlations between “gross motor” and the overall score of the four questionnaires were not as strong, varying between .46 and .58, with the exception of Q48, with a correlation of .74.

**Table 3:** Correlations between developmental domains and overall scores for Mohawk and U.S. populations

**Age interval**	**n**		**Communication**		**Gross motor**		**Fine motor**		**Problem solving**		**Personal and social**	
	**Mo**	**U.S.**	**Mo**	**U.S.**	**Mo**	**U.S.**	**Mo**	**U.S.**	**Mo**	**U.S.**	**Mo**	**U.S.**
36 months^a^	68	248	.77	.77	.48	.77	.79	.78	.49	.83	.73	.73
42 months^b^	55	956	.64	.82	.58	68	.73	.82	.80	.84	.64	.80
48 months^a^	74	336	.87	.73	.74	.69	.82	.82	.79	.66	.76	.75
54 months^b^	61	590	.75	.81	.46	.68	.87	.81	.62	.75	.81	.77

*Note.* Mo: Mohwak. All correlations are significant at *p* < .01 except for the U.S. population^b^ at *p* < .0001.

Correlations for the U.S. sample were combined from two different sources: ^a^Squires et al. [3] (second edition) and ^b^Squires et al. [18] (third edition) user’s guides as the coefficients for the 42- and 54-month intervals were not available in the second edition. Coefficients for the U.S. sample are presented for informational purposes.

The same procedure that was used with the U.S. normative study [22] was also used to calculate referral cut-off points. Two standard deviations were subtracted from the mean score of each developmental domain. Table 4 presents means, standard deviations and cut-off points by questionnaire for each developmental domain, comparing the Mohawk and U.S. populations. Out of 20 mean comparisons, 8 were statistically significant (communication 42- and 54-mo; gross motor 36- and 54-mo; fine motor 36- and 42-mo; problem solving 36- and 48-mo). However, none of these had a raw score difference greater than 5, which is the smallest scoring increment on the ASQ (items may be scored 0, 5, or 10 points). The Mohawk cut-off point was higher for all domains except for “fine motor” 48- and 54-mo and “problem solving” 54-mo. However, standard deviations were lower in the Mohawk population, which suggests lesser variability in the sample.

**Table 4:** Comparison of Mohawk and U.S. cut-off points and means for ASQ developmental domain scores

**Age**	**Sample**	**n**	**Communication**			**Gross motor**			**Fine motor**			**Problem solving**			**Personal and social**		
			**M**	**SD**	**CP**	**M**	**SD**	**CP**	**M**	**SD**	**CP**	**M**	**SD**	**CP**	**M**	**SD**	**CP**
36	Mo	68	53.75	7.65	38.45	56.46*	4.97	46.52	50.46*	11.40	27.66	55.26*	5.82	43.62	53.24	6.80	39.64
	U.S.	1007	51.88	10.44	30.99	54.68	8.84	36.99	47.07	14.50	18.07	51.97	10.84	30.29	52.82	8.74	35.33
42	Mo	55	53.27*	7.15	38.97	55.69	6.35	42.99	50.35*	9.53	31.29	55.86*	6.82	42.22	52.33	6.57	39.19
	U.S.	956	50.02	11.48	27.06	54.03	8.88	36.27	47.55	13.87	19.82	51.54	11.72	28.11	51.39	10.13	31.12
48	Mo	74	53.43	9.82	33.79	54.55	7.72	39.11	45.08	15.01	15.06	52.97	9.36	34.25	52.23	9.80	32.63
	U.S.	672	52.92	11.10	30.72	52.71	9.97	32.78	45.35	14.77	15.81	52.78	10.74	31.3	50.34	11.87	26.60
54	Mo	61	56.07*	5.92	44.23	57.58*	4.17	49.24	45.67	11.83	22.01	50.70	9.31	32.08	54.02	7.63	38.76
	U.S.	590	53.79	10.97	31.85	53.98	9.40	35.18	46.12	14.40	17.32	51.25	11.56	28.12	52.77	10.22	32.33

*p < .05.

*Note.* Mo: Mohawk, CP: Cut-off Point. The data related to the U.S. sample are available in the *ASQ-3 User’s Guide*[18].

Objective 2 is to describe the agreement between parents and teachers with respect to referring the child for further evaluation according to the cut-off’s from the original ASQ. Of the 394 questionnaires completed by both parents and teachers, results from parents suggested referral for more in-depth evaluation for 41 children, whereas results from teachers suggested referral for 74. In evaluating inter-rater reliability, ASQ results (i.e., scores above or below cut-off) of parents and teachers did not agree for 59 (15%) of the children, however of these cases 76% (n = 45) had results that were in accordance for four of the five domains (Table 5). For results that exhibited disagreement, over a third occurred in the communication domain (n = 25) and nearly a quarter occurred in the problem solving domain (n = 18).

**Table 5:** Number of domains where there are disagreements between parents and teachers

**Age interval**	**n children**	**% children parent-teacher disagreement**	**n domains disagreement**			
			**1 domain**	**2 domains**	**3 domains**	**4 domains**
10 months	3	.3% (n = 1)	0	1	0	0
12 months	4	0% (n = 0)	0	0	0	0
14 months	4	.5% (n = 2)	2	0	0	0
16 months	4	0% (n = 0)	0	0	0	0
18 months	8	.3% (n = 1)	1	0	0	0
20 months	10	1.5% (n = 6)	5	1	0	0
22 months	8	.3% (n = 1)	0	1	0	0
24 months	14	0% (n = 0)	0	0	0	0
27 months	23	1% (n = 4)	2	2	0	0
30 months	25	1.8% (n = 7)	4	2	1	0
33 months	23	.8% (n = 3)	3	1	0	0
36 months	62	2.5% (n = 10)	8	2	0	0
42 months	53	1.3% (n = 5)	5	0	0	0
48 months	74	2.3% (n = 9)	6	3	0	0
54 months	61	1.3% (n = 5)	4	1	0	0
60 months	18	1.3% (n = 5)	5	0	0	0
Total	394	15% (n = 59)	45	13	1	0

*Note.* Children were evaluated more than once but with different age intervals of ASQ.

Objective 3 is to explore whether the ASQ is culturally appropriate for the First Nations community. The research team conducted a focus group with parents to gather more information about their experience with the ASQ in terms of its user-friendliness, the time required to complete it, the availability of the materials needed, the relevance of the items to the Mohawk culture, and its usefulness.

In general, the ASQ was described by parents as a fun to complete and easy to use. Parents reported that they felt it helped them to become more aware of their child’s abilities. However, some parents found the rating procedure confusing, especially when it came to making a distinction between the “sometimes” and “yes” response options. They suggested that checklists specifying needs (including examples) should be created to help choose between “sometimes” or “yet”. Parents also suggested that adding more visual cues, pictures and symbols could help their comprehension of some of the items. They also felt that it is important for parents to understand that it is normal for a child to be unable to accomplish all items on the questionnaire and that it is important not to focus on mistakes or “wrong” answers.

Note, although the visual modifications to the ASQ were made before starting the study, the other modifications that addressed changes to clarify or adapt item appraisal were made during the study in response to feedback from parents. For example, in Q48 and Q54, for the item “Does your child color mostly within the lines?” we provided a drawing to clarify “mostly within the lines”. In the Q36 and Q42 details were added to clarify scoring criteria for the item “When you ask, ‘What is your name?’ does your child say both her first and last name?” so that a “yes” response is selected if a child can say his single Mohawk name. This modification to the item was made to reflect cultural norms as it is culturally acceptable to state only a first name when using a Mohawk name. Furthermore, in Mohawk communities, home addresses are not used. Instead, people provide descriptive statements about where they live. As a result, adaptations may be required to so that ASQ items are more reflective and congruent with the cultural practices and logistic realities of this and other particular populations.

In relation to testing conditions for younger children, several attempts were sometimes necessary in order to encourage the children to attempt or to perform some of the activities that do not typically occur. In some instances events were also staged in order to elicit and observe some of the skills. Parents suggested creating a strategy sheet to show them how to perform or stage the activities. Regarding materials, most parents had the required toys and tools except for child-friendly scissors. They suggested creating a take-home kit for parents who need it and providing ideas for alternative materials that could be used. In the end however, developmental screening with the ASQ was generally considered a good idea, and an important one for early intervention, and parents communicated support of its continued use in their child’s preschool program.

## Discussion

The purpose of this study was to assess the relevance and usefulness of the ASQ for the parents of Mohawk children and to collect the data needed to validate the tool within this context.

Objective 1 focused on the internal consistency indices (Cronbach’s alpha, correlations and cut-off points). In general, the alpha coefficients from the Mohawk sample reflected acceptable internal consistency. However, five results, in Q36 and Q42, reflected low alpha coefficients (< .60): “gross motor” (Q36 and Q42), “problem solving” (Q36), and “personal-social” (Q36 and Q42). In both the U.S. [22] and Chinese [23] versions, Q36 also presented low internal consistency coefficients in the “personal-social” domain. According to Tsai et al. [23], it is possible that the items composing “personal-social” evaluate two different domains instead of just one. However, even when we removed one item, the increased alpha values for the five domains were still not sufficient. As for the correlations between developmental domains and the total score, they were generally good. However, three were low (< .50), specifically “gross motor” (Q36 and Q54) and “problem solving” (Q36). These low correlations between developmental domain and total score may be related to the fact that in the within domain analyses the coefficient alphas were also low suggesting weak internal consistency. Similarly to the items in the personal-social domain, it is possible that the items in gross motor Q36 and Q54 address many different types of skills (e.g., jumping, climbing stairs, kicking a ball, and throwing a ball). The analysis reveals few significant differences between the U.S. and Mohawk populations in terms of mean developmental domain scores, which suggests a similarity between the two populations. These results confirm those of Jason and Squires [18] with Norwegian and American populations and those of Heo et al. [20] with Korean and American children. Differences in four of five developmental domains were observed (communication, gross motor, fine motor and problem solving). For all of these, Mohawk children had higher scores than their American peers. It is possible that certain abilities are acquired at different developmental periods depending on the country of origin (e.g. unbutton one or more buttons; name numbers). In addition, contrary to the American population, the Mohawk sample was composed only of children enrolled in a preschool where an educational program was implemented.

Objective 2 analyzed the agreement between parents and teachers regarding referring the child for further evaluation (i.e., scores above or below cut-offs). When comparing the results determining whether a child’s score indicates typical development or whether he or she should be referred for a more comprehensive assessment, parents and teachers had a generally good agreement ratio. The greatest rates of disagreement were found in the “communication” and “problem solving” domains. It is possible that differences pertaining to expectations in family and preschool settings could influence the interpretation of a successful response to an item (i.e., Q36-C1: When you ask, “What is your name?” does your child say both her first and last names?; Q27-PS : If your child wants something he cannot reach, does he find a chair or box to stand on to reach it?) and/or that many parents were unsure how to rate some of the items “sometime” or “yes”.

Objective 3 explored whether the ASQ is culturally appropriate for the First Nations community. Regarding the evaluation of the ASQ by its users, the results were similar to those observed by users of the Quebec French version. As reported by the Dionne et al. [19] study in Quebec, and by Ball [9] in Western Canada, users consider the ASQ to be an easy, simple, straight-forward and pleasant tool to use, that also facilitates discussions with parents. However, several Mohawk parents mentioned having difficulties discriminating between “sometimes” and “yes” response options. They also suggested adding visual supports (drawings), and providing examples of strategies that would help their child complete the activities targeted on the questionnaires.

This study has certain limitations. First, the sample was small. A larger sample in all ASQ intervals would help to investigate the applicability of the questionnaires with Mohawk children. In addition, the family income was relatively high and not representative of a normal distribution of incomes in the community.

Although this study with a Mohawk community offers useful and pertinent material for reflection, the conclusions cannot be generalized to all First Nation peoples, since all the children were enrolled in preschool, in a community located near a large urban area (Montreal). However, the results may be useful in determining principles to be used in judging the adequacy of a tool for use with a particular culture.

It is important to examine the results of this study in relation to the type of assessment studied—a screening tool. Items are usually selected in accordance with the typical performance of same-age children [24] and according to developmental markers that make it possible to establish whether the child has a typical developmental pattern or not. In the absence of developmental standards for young First Nation children, it is difficult to identify the skills that can be used as developmental markers or milestones. However, studying screening tests like the ASQ may help in establishing normative information for specific populations.

In addition, one attractive quality of screening tests is their ability to be used by parents and caregivers with little specific training [25]. It is interesting to note that caregivers and parents could fill out the screening questionnaires in our study without any formal training. In our context, it was of the outmost importance to involve parents in the screening process given their knowledge of the First Nation culture.

## Conclusions

Preliminary results of the present study indicate that the ASQ is an appropriate tool for the Mohawk community. It is critical that we continue to investigate the adequacy of assessment tools to be used with Canadian First Nation populations. There are plans to replicate this study with a larger number of children from the same community, as well as with other First Nation populations. However, the need to sustain and develop culturally appropriate assessments should not merely result in the use of existing tools. A discussion forum on the development of assessment tools by various First Nation communities might be another avenue worth investigating. Indeed, the diversity of these peoples’ physical, human and social environments raises the question of the appropriateness of having tools adapted to these communities as a whole.

In regards to screening tools in particular, another path to explore may be the use of tools that include items less sensitive to cultural influence. In this regard, the ASQ remains a screening test that may be appropriate for use with children from communities that are seemingly very different in terms of geographic, climatic and cultural backgrounds. To date, the ASQ has been translated and adapted for use with several different populations and languages with apparent success. This preliminary study with the Child and Family Center appears to support further study and the use of the ASQ with the Mohawk population.

## Abbreviations

ASQ: Ages & stages questionnaires.

## Competing interests

The study was funded by a grant from the Social Sciences and Humanities Research Council (SSHRC). The funder did not have any role in the study design, analysis, interpretation or dissemination of research findings. The authors declare that they have no competing interests.

## Authors’ contributions

All authors contributed to the conceptualization and design of study. CD and SM drafted the manuscript. SM performed the statistical analysis. CD and SM interpreted the data. CD, SM, JS and JC revised the manuscript. All authors read and approved the final manuscript.

## Pre-publication history

The pre-publication history for this paper can be accessed here:

http://www.biomedcentral.com/1471-2431/14/23/prepub
